# Chromothripsis Is a Recurrent Genomic Abnormality in High-Risk Myelodysplastic Syndromes

**DOI:** 10.1371/journal.pone.0164370

**Published:** 2016-10-14

**Authors:** María Abáigar, Cristina Robledo, Rocío Benito, Fernando Ramos, María Díez-Campelo, Lourdes Hermosín, Javier Sánchez-del-Real, Jose M. Alonso, Rebeca Cuello, Marta Megido, Juan N. Rodríguez, Guillermo Martín-Núñez, Carlos Aguilar, Manuel Vargas, Ana A. Martín, Juan L. García, Alexander Kohlmann, M. Consuelo del Cañizo, Jesús M. Hernández-Rivas

**Affiliations:** 1 Unidad de Diagnóstico Molecular y Celular del Cáncer, Centro de Investigación del Cáncer-IBMCC (USAL-CSIC), Salamanca, Spain; 2 IBIOMED, Instituto de Biomedicina, Universidad de León, León, Spain; 3 Servicio de Hematología, Hospital Universitario de León, León, Spain; 4 Servicio de Hematología, Hospital Universitario de Salamanca, Salamanca, Spain; 5 Servicio de Hematología, Hospital Jerez de la Frontera, Cádiz, Spain; 6 Servicio de Hematología, Hospital Río Carrión, Palencia, Spain; 7 Servicio de Hematología, Hospital Clínico Universitario de Valladolid, Valladolid, Spain; 8 Servicio de Hematología, Hospital del Bierzo, Ponferrada, Spain; 9 Servicio de Hematología, Hospital Juan Ramón Jiménez, Huelva, Spain; 10 Servicio de Hematología, Hospital Virgen del Puerto, Plasencia, Spain; 11 Servicio de Hematología, Hospital General de Soria, Soria, Spain; 12 Servicio de Hematología, Hospital Comarcal de Jarrio, Jarrio-Coaña, Spain; 13 AstraZeneca, Personalized Healthcare and Biomarkers, Innovative Medicines and Early Development, Cambridge, United Kingdom; 14 IBSAL, Instituto de Investigación Biomédica de Salamanca, Salamanca, Spain; University of Navarra, SPAIN

## Abstract

To explore novel genetic abnormalities occurring in myelodysplastic syndromes (MDS) through an integrative study combining array-based comparative genomic hybridization (aCGH) and next-generation sequencing (NGS) in a series of MDS and MDS/myeloproliferative neoplasms (MPN) patients. 301 patients diagnosed with MDS (n = 240) or MDS/MPN (n = 61) were studied at the time of diagnosis. A genome-wide analysis of DNA copy number abnormalities was performed. In addition, a mutational analysis of *DNMT3A*, *TET2*, *RUNX1*, *TP53* and *BCOR* genes was performed by NGS in selected cases. 285 abnormalities were identified in 71 patients (23.6%). Three high-risk MDS cases (1.2%) displayed chromothripsis involving exclusively chromosome 13 and affecting some cancer genes: *FLT3*, *BRCA2* and *RB1*. All three cases carried *TP53* mutations as revealed by NGS. Moreover, in the whole series, the integrative analysis of aCGH and NGS enabled the identification of cryptic recurrent deletions in 2p23.3 (*DNMT3A*; n = 2.8%), 4q24 (*TET2*; n = 10%) 17p13 (*TP53*; n = 8.5%), 21q22 (*RUNX1*; n = 7%), and Xp11.4 (*BCOR*; n = 2.8%), while mutations in the non-deleted allele where found only in *DNMT3A* (n = 1), *TET2* (n = 3), and *TP53* (n = 4). These cryptic abnormalities were detected mainly in patients with normal (45%) or non-informative (15%) karyotype by conventional cytogenetics, except for those with *TP53* deletion and mutation (15%), which had a complex karyotype. In addition to well-known copy number defects, the presence of chromothripsis involving chromosome 13 was a novel recurrent change in high-risk MDS patients. Array CGH analysis revealed the presence of cryptic abnormalities in genomic regions where MDS-related genes, such as *TET2*, *DNMT3A*, *RUNX1* and *BCOR*, are located.

## Introduction

The progressive accumulation of genetic aberrations such as copy number abnormalities, in the form of gains or losses of genetic material affecting certain regions of the genome, and particular gene mutations, provide the basis for cancer development [1,2]. However, recent studies have revealed the presence of an alternative mechanism, termed chromothripsis, in which massive chromosome rearrangements occur in a one-step catastrophic event, indicating that chromosome instability is a central aspect of cancer cell biology. A key feature of chromothripsis is the occurrence of tens to hundreds of clustered genomic rearrangements usually in one or, in some instances, several chromosomes. This complex abnormality can affect an entire chromosome, a chromosome arm, or be confined to a single region of a chromosome [2–9]. These rearrangements usually appear crisscrossing the involved regions [4], and chromosomes affected by chromothripsis show a characteristic pattern of copy number oscillations between two (or occasionally three) copy number states [4,8]. By far the simplest explanation of such rearrangements is that, at some stage during cancer development, distinct chromosomes or chromosomal regions are broken into many segments and then inaccurately stitched back together by DNA repair mechanisms [2,7,10]. Chromothripsis was initially observed in chronic lymphocytic leukemia (CLL), but it is present in a wide range of human cancers, including multiple myeloma (MM), acute lymphoblastic leukemia (ALL), acute myeloid leukemia (AML), Hodgkin lymphoma, bone cancers, medulloblastoma, neuroblastoma, colorectal cancer and melanoma. Some congenital disorders also show chromothripsis [3–17].

Myelodysplastic syndromes (MDS) are a clinically heterogeneous group of clonal hematopoietic stem cell disorders characterized by morphological dysplasia, ineffective hematopoiesis and peripheral blood cytopenias [18]. MDS and chronic myelomonocytic leukemia (CMML), an entity sharing features of myelodysplastic syndromes and chronic myeloproliferative neoplasms (MPN) [19], have a highly variable clinical course [20]. The presence of chromosomal abnormalities is a recurrent hallmark of both MDS and CMML patients, with consequences for their diagnosis, risk stratification and prognosis [18,21,22]. In fact, these genetic changes are directly incorporated into the Revised International Prognostic Scoring System (IPSS-R) for MDS and the CMML-Specific Prognostic Scoring System (CPSS) [21,22]. In addition, gene mutations are also very frequent (80–90%) in MDS and related myeloid neoplasms [23,24]. These mutations affect transcription factors such as *RUNX1* and *BCOR*, epigenetic modulators such as *TET2*, *DNMT3A*, *IDH1/2*, *ASXL1* and *EZH2*, tumor suppressor genes such as *TP53*, several components of the RNA-splicing machinery such as *SF3B1*, *SRSF2*, *U2AF1*, and *ZRSR2*, genes involved in DNA replication such as *SETBP1*, and genes of the cohesin complex such as *STAG2*, *RAD21*, *SMC1A*, and *SMC3*. The list of genes carrying mutations involved in the pathogenesis of MDS is still growing [18,23–25].

Conventional metaphase cytogenetics (CC) is still the gold standard for karyotypic studies; however, diagnosis and prognostication may be difficult in the 10–15% of patients with non-informative cytogenetics, due to the absence of mitosis, or the 40–60% with a normal karyotype [26,27]. Additionally, the presence of complex karyotypes with three or more chromosomal abnormalities may hinder the identification of the chromosomes involved in these changes [28]. Therefore, these CC techniques are not sufficient for a thorough study of these myeloid malignancies. The use of molecular genome-wide scanning techniques allows the identification of cryptic abnormalities in patients with a normal karyotype and the better characterization of unbalanced genetic changes [23,24,26,27,29,30]. Thus, array-based karyotyping revealed MDS and related myeloid neoplasms with a normal karyotype to have one or more genomic abnormalities, including deletions of *TET2* and *RUNX1* genes (40%) [26,27,29]. These studies suggest the potential clinical utility of such genome-wide scanning techniques for the management of MDS and related diseases.

To gain insight into the characterization of the molecular changes in MDS and to explore novel genetic abnormalities occurring in these disorders, an integrative study combining array-based comparative genomic hybridization (aCGH) and next-generation sequencing (NGS) in a series of MDS and MDS/MPN patients was carried out. The results demonstrated the presence of infrequent but recurrent chromothripsis involving chromosome 13 in these diseases and also showed that aCGH could be used in a clinical setting as a complementary method to conventional cytogenetics for identifying copy number changes in MDS and CMML patients.

## Materials and Methods

### Patients

A total of 301 patients diagnosed with MDS (n = 240) or MDS/MPN (n = 61) at the time of diagnosis were studied. The main clinical characteristics of the patients are summarized in Table 1. The median age was 77 years (range, 11–93 years), and 191 patients (63.5%) were male. Diagnoses were established according to the 2008 World Health Organization criteria [31] (Table 1). This study was performed in accordance with the Declaration of Helsinki guidelines, and was approved by the Local Ethical Committees “Comité Ético de Investigación Clínica, Hospital Universitario de Salamanca”. All patients provided written informed consent.

**Table 1:** Main characteristics of the whole series of patients included in the study.

Variables		Median [Range]
**Number of patients**		301
**Gender** (Male/Female)		191 / 110
**Age** (years)		77 [11–93]
**Peripheral blood values**		
	Hemoglobin level (g/dl)	10 [4–17]
	Neutrophil count (x10^9^/L)	2.5 [0.1–90]
	Platelet count (x10^9^/L)	125 [5–1018]
**Bone marrow blasts (%)**		1.4 [0–20]
**WHO 2008 classification**		
**MDS**		**240**
	RCUD	20
	RARS	11
	RCMD	147
	RAEB-1	23
	RAEB-2	23
	MDS-U	9
	MDS del(5q)	7
**MDS/MPN**		**61**
	CMML-1	51
	CMML-2	7
	RARS-T	3
**Conventional cytogenetics**		
**Normal**		**216**
	≤ 10 metaphases	14
	11–19 metaphases	38
	≥ 20 metaphases	164
**Abnormal**		**45**
	-5/del(5q)	9
	double including del(5q)	1
	-7/del(7q)	1
	+ 8	3
	del(11q)	2
	del(20q)	1
	-Y	8
	complex (≥3 abnormalities)	12
	any other single abnormality	8
**Non-informative**		**40**

Abbreviations: WHO, World Health Organization; MDS, myelodysplastic syndromes; RCUD, refractory cytopenia with unilineage dysplasia; RARS, refractory anemia with ringed sideroblasts; RCMD, refractory cytopenia with multilineage dysplasia; RAEB, refractory anemia with excess of blasts; MDS-U, MDS unclassified; MDS del(5q), MDS associated with isolated del(5q); MDS/MPN, myelodysplastic/myeloproliferative neoplasms; CMML, chronic myelomonocytic leukemia; RARS-T, RARS with thrombocytosis; del, deletion.

### Array-based comparative genomic hybridization studies

Genome-wide DNA copy number abnormalities (CNAs) were analyzed in bone marrow samples from all patients using the Human CGH 12x135K Whole-Genome Tiling v3.0 Array (Roche NimbleGen, Madison, WI, USA). For sample preparation and hybridization the NimbleGen CGH array standard protocol was followed [32] (See S1 Methods). All detected CNAs were carefully reviewed to identify regions overlapping those previously reported to be copy number variants (CNVs) in the Database of Genomic Variants (http://dgv.tcag.ca/); these were excluded from subsequent analysis. Genomic abnormalities were interpreted and reported in accordance with the International System for Human Cytogenetic Nomenclature (ISCN 2013) guidelines [33]. All the array data discussed in this publication have been deposited in NCBI's Gene Expression Omnibus and are accessible through GEO Series accession number GSE67682.

Additionally, conventional metaphase cytogenetic results were available from all patients. Based on CC results, patients were divided into three groups: patients with non-informative cytogenetics (n = 40, 13.3%) due to the absence of mitosis, patients with an abnormal karyotype (n = 45), and patients with a normal karyotype (n = 216, 71.7%). The latter group was further divided into three categories according to the number of good-quality metaphases evaluated: ≥20 metaphases (n = 164), between 11 and 19 metaphases (n = 38), and ≤10 metaphases (n = 14). Detailed information about the cytogenetic groups is summarized in Table 1, and the cytogenetic abnormalities found in the whole series are listed in S1 Table.

All genomic changes found by aCGH but not detected by conventional metaphase cytogenetics were validated by interphase fluorescence *in situ* hybridization (FISH), in the case of large recurrent deletions and gains, or by using an independent genome-wide analysis of DNA copy number changes with the SurePrint G3 Human CGH Microarray (8x60k) (Agilent Technologies, Palo Alto, CA, USA) for small recurrent and individual abnormalities.

### Next-generation sequencing studies

Mutations in *DNMT3A*, *TET2*, *RUNX1*, *TP53* and *BCOR* genes were screened by amplicon-based next-generation sequencing (NGS) in selected cases using 454 Titanium amplicon chemistry (454 Life Sciences, Branford, CT, USA). Briefly, all coding exons of *TET2*, *RUNX1* and *BCOR*, exons 7–23 of *DNMT3A* and exons 4–11 of *TP53* were covered by 27, 7, 29, 16 and 8 amplicons, respectively (S3 Table). Amplicon libraries were prepared following the manufacturer’s recommendations and previously described methods [34] (See S1 Methods).

For the detection of variants, all amplicon reads were analyzed with the Sequence Pilot software (v3.5.2; JSI medical systems, Kippenheim, Germany) and GS Amplicon Variant Analyzer Software (v2.9; 454 Life Sciences). Single nucleotide polymorphisms (SNPs) (http://www.ncbi.nlm.nih.gov/SNP/) and variants within introns were not scored. In addition, all mutations were validated by resequencing of PCR products from new independent PCRs.

## Results

### Chromothripsis on chromosome 13 is a recurrent abnormality in high-risk MDS

**Fig 1:**
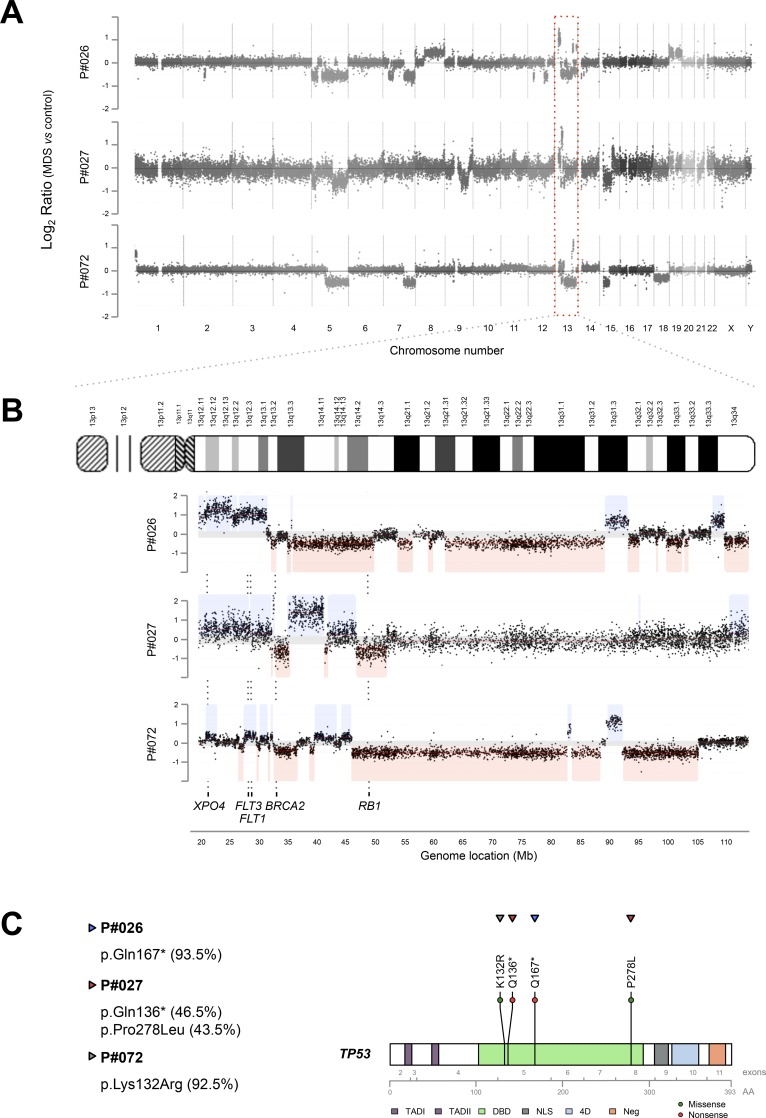
Recurrent chromothripsis on chromosome 13 in high-risk MDS. (**A**) Whole genome view ratio plots derived from aCGH data of MDS patients (#026, #027 and #072) showing chromothripsis on chromosome 13, indicated by the red-shaded box. The three patients had complex karyotypes: patient #026 had 33 aberrations and affecting seven chromosomes; patient #027 had 19 abnormalities affecting five chromosomes; patient #072 had 21 aberrations affecting six chromosomes. The Y-axis represents the log_2_ ratio values of MDS:control signal intensities for each probe. The X-axis illustrates all the probes in the array sorted by chromosome and physical mapping position. Chromosome numbers are indicated below the X-axis. (**B**) Detailed view of the whole chromosome 13 in patients #026, #027 and #072 showing a complex pattern of alternating copy number gains and losses. The grey area represents the thresholds of signal values (log_2_ ratio) to call CNAs, red lines indicate segmented copy number profiles, and boxes shaded in pale-red and pale-blue depict copy number losses and gains, respectively. Patient #026 had 18 alternating copy number changes, patient #027 had 11, and patient #072 had 16 changes along the whole chromosome 13. Copy number profiles differed between these patients. The Y-axis represents log_2_ ratios and the X-axis shows all probes of chromosome 13 sorted by chromosome position. Genomic location (Mb) is indicated below the X-axis. (**C**) Distribution of *TP53* mutations identified by amplicon-based deep sequencing in the three MDS patients with chromothripsis. All *TP53* mutations were located in the sequence-specific DNA binding domain. One patient had two mutations in heterozygosis, while the other two patients had one mutation each in homozygosis. The variant allele frequencies (VAFs) are represented in brackets. Each circle represents a mutation found in one patient. Green and red circles depict missense and nonsense mutations, respectively. Each patient is illustrated by a different-colour triangle. The complete coding region of *TP53* is illustrated and the respective exons and amino acid (AA) positions are indicated at the bottom. The following protein domains are shown: TAD1 and TAD2, amino-terminal transactivation domains 1 and 2; DBD, sequence-specific DNA-binding domain; NLS, nuclear localization signalling domain; 4D, carboxy-terminal tetramerization domain; Neg, negative regulation domain. Gene variants were represented using the R package “R453PlusToolbox” [53].

The analysis of copy number profiles derived from aCGH data identified complex genomic rearrangements on the aCGH chromosome plots, showing multiple non-contiguous CNAs, with the hallmarks of chromothripsis. Based on previous studies, evidence of chromothripsis was defined as the presence of at least ten changes in segmental copy number between two or three copy number states on an individual chromosome [6]. Using these criteria, three MDS cases (1.2%) exhibited chromothripsis (Fig 1A and 1B), with more than 11 copy number changes involving exclusively chromosome 13. The copy number states rapidly alternate between one (deletion), two (normal) and three (gain) copies. The patterns of genomic alteration were different between the three high-risk MDS patients. However, it should be pointed out that involvement of a total of 91 genes mapping on chromosome 13 were common to the three patients. As examples *XPO4*, *FLT3* and *FLT1* were commonly amplified genes; *BRCA2* and *RB1* were commonly deleted genes (S6 Table). All these results were validated using an independent microarray (SurePrint G3 Human CGH Microarray, 8x60k, Agilent Technologies). The three patients with chromothripsis were diagnosed as high-risk MDS (RAEB 3/40; 7.5%), two of them were RAEB-1, with 6% and 8% of BM blasts, respectively, and the remaining patient was RAEB-2, with 12% of BM blasts. All three patients died within one year. All of them had a complex karyotype revealed by aCGH (3/17; 17.6%; patients #026, #027, #072; Fig 1A), with a median of 21 CNAs (range, 19–33) throughout the whole genome. The three patients showed genomic losses on 5q23.2-q35.3, two of them also carried losses on 7q22.3-q36.3 and 15q11.1–21.2 (S1 Table).

All cases showing chromothripsis carried *TP53* mutations as revealed by NGS (Fig 1C). Specifically, two missense mutations (p.Lys132Arg, p.Pro278Leu) and two nonsense mutations (p.Gln136*, p.Gln167*) were identified. The p.Gln167* and p.Lys132Arg mutations, located in exon 5, were observed in one patient each with a variant allele frequency (VAF) of 93.5% and 92.5%, respectively. However, the p.Gln136* and p.Pro278Leu mutations were observed in the same patient with VAFs of 46.5% and 43.5%, and were located on exon 5 and exon 8, respectively. Taken together, most of these mutations affected exon 5 of *TP53*, and all of them were located in the sequence-specific DNA binding domain, which plays a central role in transcriptional transactivation (Table 2).

**Table 2:** Summary of mutations found in the analyzed genes.

Sample ID	Gene	Nucleotide change	AA change	Mutation type	VAF (%)	COSMIC ID
#132	*DNMT3A*	c.1961G>A	p.Gly654Asp	Missense	73.5	NA
#217	*TET2*	c.1648C>T	p.Arg550*	Nonsense	96.5	COSM41644
#033	*TET2*	c.3851C>T	p.Ser1284Phe	Missense	3.5	COSM120177
#140	*TET2*	c.5562delT	p.Leu1855Trpfs*32	Frameshift deletion	89	NA
#074	*TP53*	c.824G>A	p.Cys275Tyr	Missense	52	COSM165084
#172	*TP53*	C.583A>T	p.Ile195Phe	Missense	76.5	COSM129840
#130	*TP53*	c.821T>A	p.Val274Asp	Missense	45	COSM165076
#171	*TP53*	c.659A>C	p.Tyr220Ser	Missense	54	COSM251427
#072	*TP53*	c.395A>G	p.Lys132Arg	Missense	92.5	COSM308311
#027	*TP53*	c.406C>T	p.Gln136*	Nonsense	46.5	COSM126985
#027	*TP53*	c.833C>T	p.Pro278Leu	Missense	43.5	COSM129831
#026	*TP53*	c.499C>T	p.Gln167*	Nonsense	93.5	COSM121081

Abbreviations: AA, amino acid; VAF, variant allele frequency; COSMIC, catalogue of somatic mutations in cancer; NA, not available; Gly, Glycine; Asp, Aspartic acid; Arg, Arginine; Ser, Serine; Phe, Phenylalanine; Leu, Leucine; Trp, Tryptophan; fs, frameshift; Cys, Cysteine; Tyr, Tyrosine; Ile, Isoleucine; Val, Valine; Lys, Lysine; Gln, Glutamine; Pro, Proline

*, the indicated amino acid is changed to a stop codon.

### aCGH and NGS allow the identification of hidden recurrent genetic CNAs and gene mutations in MDS

A total of 285 abnormalities were identified (1–33 changes per patient) in 71 of the entire series of 301 patients (23.6%): 61 of 244 (25%) were MDS patients, while 10 of 58 (17.3%) had a diagnosis of CMML. Copy number losses (72.6%) were more frequent than gains (27.4%). The detected CNAs were distributed amongst all chromosomes except for chromosomes 14 and 16.

**Fig 2:**
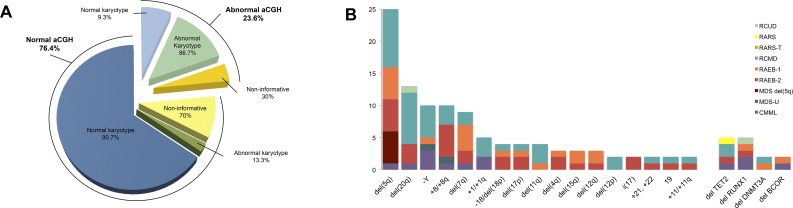
Summary of recurrent CNAs found in the global series. (**A**) Proportion of the whole series of patients with normal and abnormal aCGH profiles. Each aCGH category is then divided by the cytogenetic subgroups detected by CC studies: normal, abnormal and non-informative karyotype. Percentages represent the proportion of patients from the total number of patients within each cytogenetic subgroup. (**B**) Frequency of large recurrent genomic abnormalities and frequency of cryptic recurrent CNAs involving genes of known significance in MDS and MDS/MPN patients only seen by aCGH. All abnormalities are classified by MDS and MDS/MPN subtypes and color-coded as indicated on the right panel of the figure.

Among the global series, CNAs were present in 9.3% of the normal karyotype patients, in 86.7% of cases with abnormal cytogenetics, and in 30% of patients with unsuccessful cytogenetic analyses (Fig 2A). The most frequent large recurrent aberrations were del(5q) (35.2%), del(20q) (18.3%), -Y (14.1%), trisomy 8 (14.1%), del(7q) (12.7%), +1/+1q (7%), -18/del(18p) (5.6%), del(17p) (5.6%), del(11q) (5.6%), del(4q) (4.2%), del(15q) (4.2%), and del(12q) (4.2%) (Fig 2B). The most frequent aberrations in CMML were–Y (5.2%) and gains on 1q (3.4%), while in RCMD losses on 5q (6.1%), 20q (5.4%), and–Y (3.4%) predominated. By contrast, in RAEB patients, losses involving 5q (21.7%), 7q (13%) and 17p (6.5%), and trisomy 8 (10.9%) were frequently observed (Fig 2B). In addition, in 38 of 301 cases (12.6%), aCGH revealed the presence of 81 small aberrations (≤5 Mb), which were below the detection limit of CC. In 21 of these cases only one cryptic CNA was detected: 17 deletions and four gains. In the other 17 cases, two or more cryptic aberrations were observed, consisting of 49 deletions and 11 gains. Notably, these cryptic CNAs involved regions such as 2p23.3, 4q24, 5q33.1, 7q22.1, 21q22.12, 21q22.3 and Xp14, where genes implicated in the pathogenesis of MDS and MDS/MPN are located, including *DNMT3A*, *TET2*, *SPARC*, *CUX1*, *RUNX1*, *U2AF1* and *BCOR*, respectively (Fig 2B).

**Fig 3:**
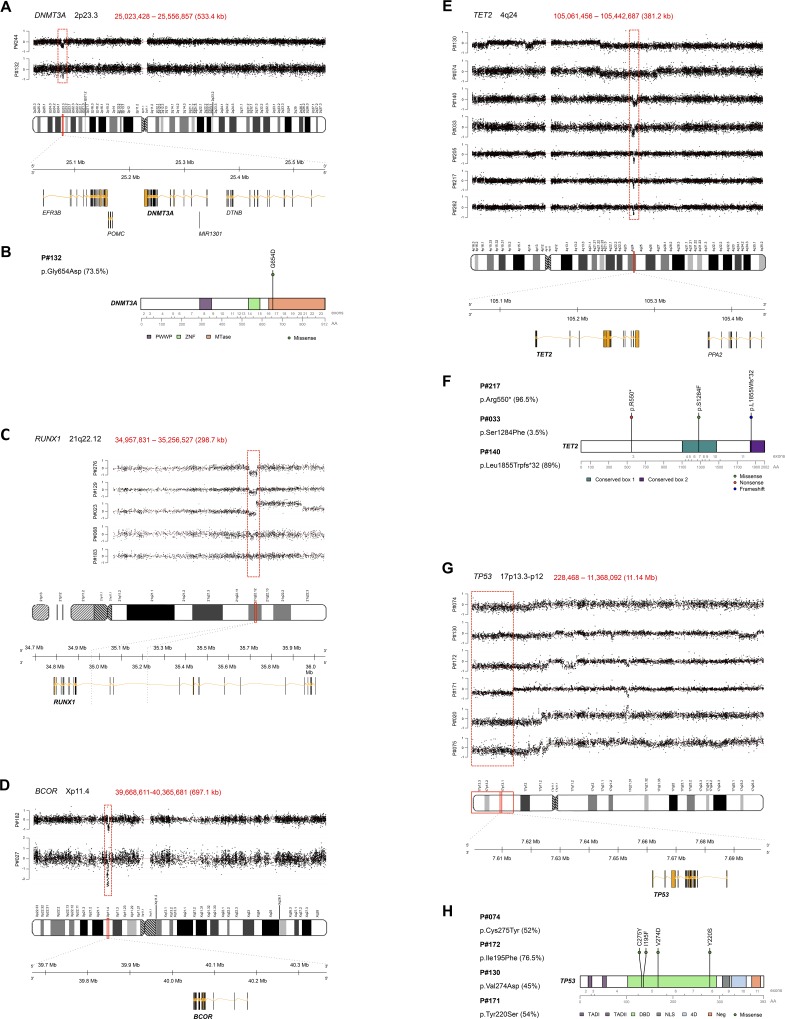
Combination of aCGH and NGS analysis for regions with frequently mutated genes in MDS and MDS/MPN. (**A**, **C**, **D**, **E**, **G**) Detailed view of the whole chromosomes 2, 4, 17, 21 and X, where recurrent regions of deletion where found by aCGH, indicated by the red-shaded box. A magnified view of the minimal deleted regions with a schematic diagram showing the genes included within the deletion. For all figures, genomic locations are indicated in Mb, and the chromosome position (bp) and size (kb) of the minimal deleted regions are indicated in the upper part of each chromosome view ratio plots. The Y-axis represents the log_2_ ratio values and all probes for each chromosome are sorted by genomic position along the X-axis. (**A**) A 533.4-kb deletion on 2p23.3 affecting the *DNMT3A* locus. (**C**) A 298.7-kb deletion on 21q22.12 affecting the *RUNX1* locus. (**D**) A 697.1-kb deletion on Xp11.4 affecting the *BCOR* locus. (**E**) A 381.2-kb deletion on 4q24 affecting the *TET2* locus. (**G**) An 11.14-Mb deletion on 17p13.3-p12 affecting the *TP53* locus. Genes were represented using the R package “GenomeGraphs”. (**B**, **F**, **H**) Distribution of *DNMT3A*, *TET2* and *TP53* mutations identified by targeted amplicon-based deep sequencing. The variant allele frequencies (VAFs) are represented in brackets. The complete coding regions of *DNMT3A*, *TET2* and *TP53* are illustrated and the respective exons and amino acid (AA) positions are indicated below. Each circle represents a mutation found in a single patient. Green, red and blue circles depict missense, nonsense and frameshift mutations, respectively. (**B**) One patient carried a *DNMT3A* missense mutation located in the MTase domain. The following protein domains are shown: PWWP, proline-tryptophan-tryptophan-proline domain; ZNF, zinc finger domain; MTase, methyltransferase domain. (**F**) Three patients with a *TET2* deletion harbored one nonsense, one missense and one frameshift mutation each. The two evolutionarily conserved domains, boxes 1 and 2, are shown. (**H**) Four patients carried one *TP53* missense mutation each. The following protein domains are shown: TAD1 and TAD2, amino-terminal transactivation domains 1 and 2; DBD, sequence-specific DNA-binding domain; NLS, nuclear localization signalling domain; 4D, carboxy-terminal tetrame-rization domain; Neg, negative regulation domain. Gene variants were represented using the R package “R453PlusToolbox” [53].

Furthermore, an in-depth analysis by NGS of the regions with genomic deletions of >100 kb by aCGH, where recurrently mutated genes in MDS and CMML are located, was carried out. Thus, aCGH analysis identified two cases with a deletion in 2p23.3 (*DNMT3A*) (Fig 3A), seven cases with a deletion in 4q24 (*TET2*) (Fig 3E), six cases with a 17p13 deletion (*TP53*) (Fig 3G), five cases with a deletion in 21q22 (*RUNX1*) (Fig 3C) and two cases with an Xp11.4 deletion (*BCOR*) (Fig 3D). NGS studies detected that one patient with a *DNMT3A* deletion carried a missense mutation (p.Gly654Asp) in the other allele with a VAF of 73.5%. This mutation was located in the methyltransferase domain (Fig 3B). Three patients harboring a *TET2* deletion carried one nonsense mutation (p.Arg550*), one missense mutation (p.Ser1284Phe) and one frameshift deletion (p.Leu1855Trpfs*32) each in the non-deleted allele. The variant allele frequencies were 96.5%, 3.5% and 89%, respectively. The latter two mutations affected the two evolutionarily conserved domains in the TET family proteins (Fig 3F). Of the six patients with a 17p13 deletion affecting the *TP53* locus, four carried one missense mutation each (p.Cys275Tyr, p.Ile195Phe, p.Val274Asp, p.Tyr220Ser) with VAFs of 52%, 76.5%, 45% and 54%, respectively. These *TP53* mutations were located in the sequence-specific DNA binding domain. None of the other studied genes showed mutations in those patients with losses in the regions of interest (Fig 3H). All mutations are summarized in Table 2.

### Relationship between cytogenetic results and aCGH data

Array CGH results were compared with the cytogenetic data from each patient. 56 of 83 chromosomal imbalances previously identified by CC were detected by aCGH. A remarkably high correlation between CC and aCGH results was observed in this series. In addition, given that patients from different groups ascertained by CC were included in the study, we decided to analyze the concordance between CC and aCGH, but considering each cytogenetic group of patients separately: the non-informative cytogenetic, normal and abnormal karyotype groups.

Focusing on the 216 patients with a normal karyotype as determined by CC, the aCGH results were in excellent agreement with the cytogenetics of those patients with ≥20 and 11–19 metaphases studied (92.1 and 89.5%, respectively). However, only 78.6% of those patients with ≤10 successful metaphases and no changes by CC displayed no copy number changes by aCGH (S1 Fig). Thus, 20 patients (9.3%) with a normal karyotype as determined by CC showed at least one genomic abnormality by aCGH. Only one chromosome was affected in 16 of these patients. Considering those patients with an aberrant karyotype, aCGH revealed the same genomic abnormalities as previously identified by CC in 86.7% of cases. Indeed, only six of the 45 (13.3%) cases with an abnormal karyotype established by G-banding analysis showed no copy number abnormalities with aCGH. One of these patients had a balanced translocation, which was not detectable by aCGH, and four patients had chromosomal abnormalities in three metaphases of the analyzed cells, clonal cell populations below the detection limit of the aCGH. Detailed information about these discordant cases is presented in S1 Table. With respect to the patients with unsuccessful cytogenetic analyses, 70% of cases displayed a normal aCGH profile, while 30% had at least one copy number aberration. Four patients (three high-risk MDS and one CMML) had a complex karyotype, defined by the presence of at least five copy number changes, as revealed by aCGH.

In addition, a correlation study between the aCGH profile and age, as well as WHO-based risk category, and risk stratification following the IPSS-R for MDS cases and the CPSS for the CMML patients was carried out. We found that age (*P* = 0.662) was not statistically associated with the presence of normal or abnormal aCGH findings, and that the presence of an abnormal aCGH profile was associated with high-risk MDS cases (*P*<0.05).

## Discussion

The presence of specific chromosomal abnormalities and genetic changes is a hallmark of MDS [18,23–25,35,36]. In this study, we analyzed a large cohort of MDS patients by integrating two genetic methodologies: array-based comparative genomic hybridization (aCGH) and amplicon-based deep sequencing (NGS). Our results demonstrated the presence of chromothripsis, inferred from aCGH profiles, as an infrequent but recurrent genomic abnormality in high-risk MDS. In addition, our results showed that the combination of these conventional and genome-wide scanning approaches enables a better characterization of MDS and related neoplasms, and provides new information that could improve the current diagnostic and treatment of these patients.

Chromothripsis is a genetic abnormality in which tens to hundreds of clustered genomic rearrangements occur in a one-step catastrophic event. [2–9]. In the present study, chromothripsis was observed in three cases of high-risk MDS (two cases of RAEB-1 and one patient with RAEB-2), two entities known to progress gradually to a more aggressive disease. The occurrence of chromothripsis in myeloid malignancies has been demonstrated in AMLs by SNP array-based copy number profiling, in which 8% of AML patients carried massive and complex rearrangements consistent with chromothripsis [6,37]. The molecular basis of this genomic chaos has been long studied in the last years. Therefore several mechanisms such as ionizing radiation, premature chromosome compaction, DNA replication stress, telomere shortening, abortive apoptosis, *TP53* mutations, micronuclei collapse, and hyperploidy formation have been suggested as implicated in this chromothripsis. [2,6–8,10,38–42]. In addition, approximately 50% of AML patients carrying *TP53* mutations and approximately 40% of complex karyotype AML patients displayed chromothripsis, while only 1% of AML with wild-type *TP53* and no cases with non-complex karyotype showed this aberration [6,37]. Moreover, it was also demonstrated that almost all medulloblastomas showing evidence of chromothripsis had *TP53* mutations [6]. These findings reinforce the link between somatically acquired *TP53* mutations and the presence of complex karyotypes with chromothripsis. It is of particular interest that the three MDS cases with chromothripsis reported in this study had complex karyotypes as revealed by aCGH and carried *TP53* mutations, a previously described association [6]. Additionally, the outcome of the three cases was poor, a feature that is in accordance with high-risk MDS patients, the presence of a complex karyotype or *TP53* mutations [18,21]. This complex genomic abnormality has not previously been comprehensively described in MDS patients, perhaps because the previous high-resolution copy number studies in MDS mainly concerned cases with a normal karyotype [23,26,27,29]. Other copy number studies in MDS with abnormal and complex karyotypes have been reported. These studies aimed to analyze the relationship of copy number to the CC data and their prognostic impact, and they did not show the presence of chromothripsis [28,30,43–45]. It should be noted that, in our study, chromothripsis was seen exclusively to affect the entire chromosome 13 in all three MDS patients. The presence of this genomic chaos restricted to a single chromosome has been described. Chromothripsis is typically found to affect different chromosomes at random, but was reported to involve chromosome 16p in 3/7 MM patients, and chromosome 21 in 5/9 iAMP21 ALL patients [5,17]. However, the presence of chromothripsis involving chromosome 13 has not been previously reported in myeloid malignancies [6,12,37,46]. In this study, chromothripsis affected only chromosome 13, with patterns of genomic alteration differing between the three high-risk MDS patients. *FLT3* was commonly amplified, while *BRCA2* and *RB1* were commonly deleted in the cases with chromothripsis. *FLT3* is an oncogene that regulates hematopoietic stem cell differentiation, proliferation and survival. *FLT3*-activating mutations were recurrently described in myeloid malignancies, mainly in AML, and are associated with poor prognosis [47]. *RB1* and *BRCA2* abnormalities play a role in the development of several cancers and are considered to be tumor suppressor genes [48,49]. In addition, two non-chromothripsis MDS cases (#074 and #126) showed CNAs affecting chromosome 13. One of these cases, diagnosed as RAEB-1, carried a deletion involving the *RB1* locus. Our results suggest the involvement of the *FLT3* gene and *BRCA2* and *RB1* inactivation in the pathogenesis of some cases of MDS.

The present study revealed the presence of cryptic abnormalities that were not targeted by FISH and that were below the threshold of resolution by conventional cytogenetics. In fact, 12.6% of MDS patients showed cryptic changes. Some of these submicroscopic CNAs involved regions with genes of known significance in MDS pathogenesis and were deletions in 2p23.3 (*DNMT3A*), 4q24 (*TET2*), 5q33.1 (*SPARC*), 7q22.1 (*CUX1*), 21q22.12 (*RUNX1*) and Xp11.4 (*BCOR*), and gains in 21q22.3 (*U2AF1*), that were detected in 19 patients [18,25,26,30]. These regions were equally likely to be involved in low- and high-risk MDS or CMML. In addition, the genes included in these recurrent cryptic deletions were further investigated by NGS to identify whether mutations occurred in the other allele. The sequencing results showed that only two of the seven cases with the *TET2* deletion carried *TET2* mutations, while only one of the two cases with losses in 2p23.3 showed *DNMT3A* mutations. Our results did not indicate any correlation between the presence of deletions and mutations in these MDS patients, which is in accordance with previously reported data [23]. Therefore, our results support the idea that conventional cytogenetic and aCGH studies could be complemented by the sequencing of multi-gene panels, which have already been described for MDS and related myeloid neoplasms, instead of single genes, in routine workflows for the study of these group of patients.

The presence of cytogenetic changes is a keystone in the prognosis of MDS and CMML patients. In fact, conventional cytogenetics is essential in most prognostic systems to stratify these hematological disorders [18,21,22]. However, in some subsets of MDS and CMML, conventional cytogenetic techniques fail to provide any results due to a lack of cell growth during culturing, and consequently some aberrations may be missed, making the diagnosis and prognostic stratification very difficult. Our study showed that 30% of karyotype failures carried genomic abnormalities revealed by aCGH. Four patients had complex karyotypes, two had trisomy 8 and one showed a del(5q), while the others showed deletions in 9p, 12q, 17p, 21q22, and -Y, and gains in 1q and 15q (S1 Table). Therefore, identifying these clinically relevant lesions is significant in patients with failed CC results. The clinical utility of SNP-A as a karyotyping tool in a series of MDS patients with unsuccessful cytogenetics has been previously demonstrated [50]. This method was also useful in those MDS patients with a normal karyotype when fewer than 20 good-quality metaphases are available for analysis. We demonstrated that in 10% of cases with 11–19 successful metaphases and in 21% of those with ≤10 harbored genomic aberrations aCGH will provide additional information that could redefine the prognostic risk of these patients, as previously suggested [51]. Thus, at least 20 metaphases need to be analyzed for a karyotype to be considered normal [52]. Therefore, the use of aCGH enabled the prognostic stratification according to the IPSS-R that could change the clinical management of this group of patients. Additionally, the presence of an abnormal aCGH profile was associated with high-risk patients.

In summary, the present report describes the presence of a high incidence of genomic changes in MDS and CMML patients by the integrative analysis of several molecular genetic methodologies. In addition to well-known copy number defects, the presence of chromothripsis involving chromosome 13 was a novel recurrent change in high-risk MDS patients. Moreover, aCGH analysis revealed the presence of cryptic abnormalities in genomic regions where MDS-related genes, such as *TET2*, *DNMT3A*, *RUNX1* and *BCOR*, are located. Thus, the integrative analysis of conventional cytogenetics, aCGH and NGS in MDS will provide a better understanding of the molecular abnormalities occurring in these patients, and could improve the clinical management of MDS. The potential diagnostic and prognostic value of these new genomic abnormalities should be studied in prospective studies.

## Supporting Information

S1 Fig Relationship between aCGH and CC studies in the normal karyotype group. Normal karyotype patients are divided into three categories on the basis of the number of good-quality metaphases evaluated: ≤10, 11–19 and ≥20. Patients with normal and abnormal aCGH results within each category are represented by different shades of blue.
(TIF)

S1 Methods Supplementary Methods. Additional information on patients, array-based comparative genomic hybridization studies and next-generation sequencing studies.
(DOCX)

S1 Table All genomic copy number changes detected by aCGH and all conventional cytogenetic information for the whole series. (XLSX)

S2 Table Ensembl gene and transcript IDs for the five genes selected for NGS. (XLSX)

S3 Table PCR primer-pair sequences for all amplicons representing *DNMT3A* (16), *RUNX1* (7), *TET2* (27), *TP53* (8) and *BCOR* (29) genes. (XLSX)

S4 Table PCR amplification mixes for NGS. All PCR reactions were prepared from a starting material of 30–60 ng of genomic DNA, according to the manufacturers’ recommendations. Amplification mixes #1, #2 and #4 were used with the FastStart High Fidelity PCR System, dNTPack (Roche Applied Science) and amplification mix #3 was used with the GC-RICH PCR System, dNTPack (Roche Applied Science).
(XLSX)

S5 Table PCR protocols for NGS. All PCR reactions were performed in the 96-Well GeneAmp® PCR System 9700 (Applied Biosystems, Foster City, CA).
(XLSX)

S6 Table Details of chromosome 13 rearrangements in the three high-risk MDS patients affected by chromothripsis. (XLSX)
