# Supplementary figures and images for: Chromothripsis Is a Recurrent Genomic Abnormality in High-Risk Myelodysplastic Syndromes
Source: PLoS One. 2016 Oct 14;11(10):e0164370. doi: 10.1371/journal.pone.0164370 (PMC5065168; doi:10.1371/journal.pone.0164370)

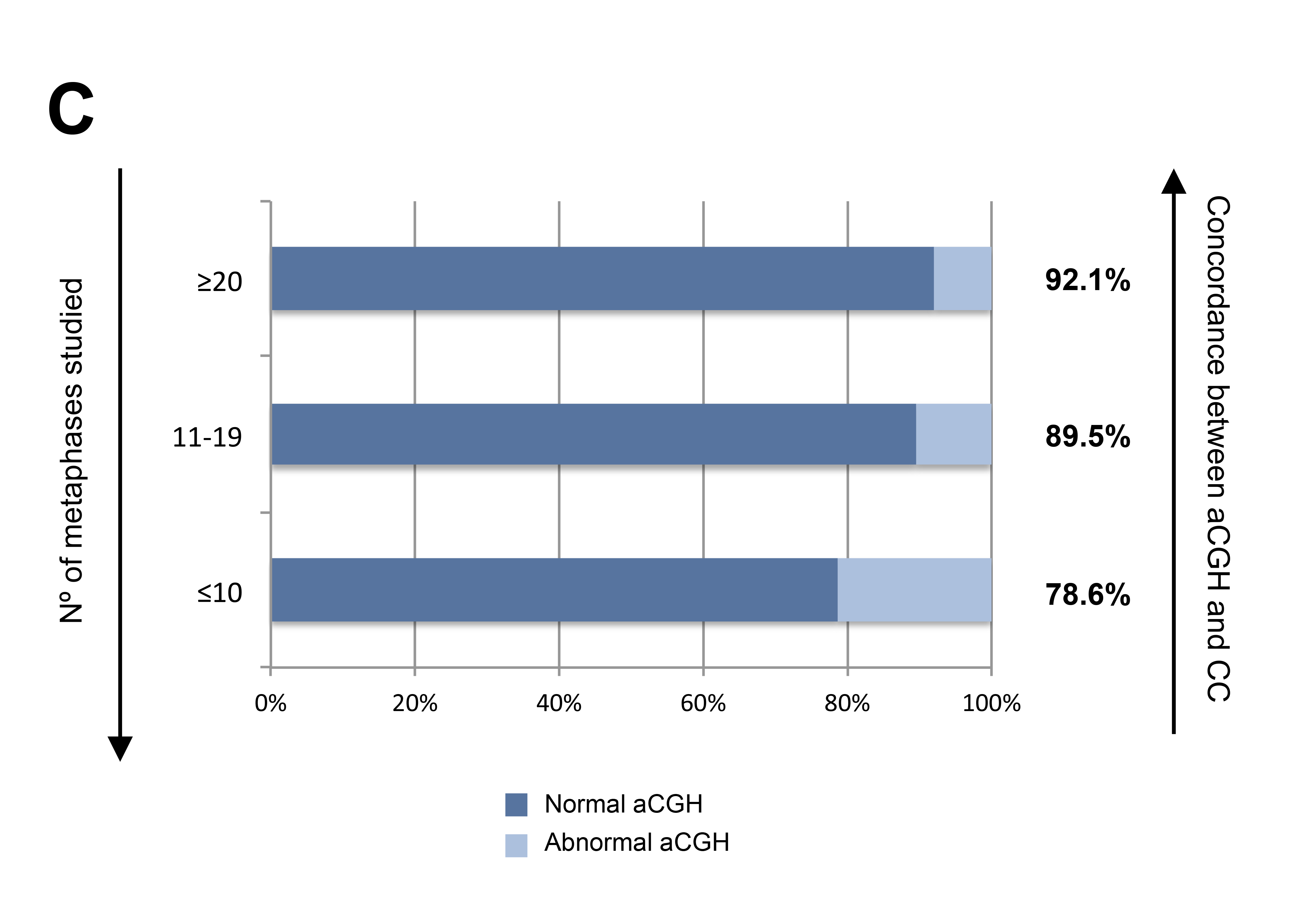

Supplement: S1 Fig — Normal karyotype patients are divided into three categories on the basis of the number of good-quality metaphases evaluated: ≤10, 11–19 and ≥20. Patients with normal and abnormal aCGH results within each category are represented by different shades of blue. (TIF) [file pone.0164370.s001.tif]
