# Supplementary material for: Chromothripsis Is a Recurrent Genomic Abnormality in High-Risk Myelodysplastic Syndromes
Source: PLoS One. 2016 Oct 14;11(10):e0164370. doi: 10.1371/journal.pone.0164370 (PMC5065168; doi:10.1371/journal.pone.0164370)
Supplement: S1 Methods — Additional information on patients, array-based comparative genomic hybridization studies and next-generation sequencing studies. (DOCX) [file pone.0164370.s002.docx]

**Supplementary Methods**

**Patients**

All patients were classified according to the 2008 World Health Organization criteria [1]. The following morphological subtypes were included: MDS associated with isolated del(5q) (MDS del(5q), n=7), refractory cytopenia with unilineage dysplasia (RCUD, n=20), refractory cytopenia with multilineage dysplasia (RCMD, n=147), refractory anemia with ringed sideroblasts (RARS, n=11), refractory anemia with excess of blasts type 1 (RAEB-1, n=23), RAEB-2 (n=23), MDS unclassified (MDS-U, n=9), CMML (n=58), and RARS with thrombocytosis (RARS-T, n=3) (Table 1).

**Array-based comparative genomic hybridization studies**

Genomic DNA (gDNA) was isolated using the QIAamp DNA Mini Kit (Qiagen, Hilden, Germany) according to the manufacturer’s standard protocol. gDNA concentration and quality were assessed with a NanoDrop spectrophotometer (ND-1000; NanoDrop Technologies, Wilmington, DE, USA) by measuring the ratio of absorbance at 260 and 280 nm (A_260_/A_280_), and also at 230 nm (A_260_/A_230_). gDNA integrity (degradation) was evaluated by running a 1% agarose gel with ethidium bromide visualization.

DNA copy number abnormalities (CNAs) were studied in all samples with the Human CGH 12x135K Whole-Genome Tiling v3.0 Array (Roche NimbleGen, Madison, WI, USA). This platform contains around 135,000 60-mer oligonucleotide probes, with a median probe spacing of approximately 12 kb (12,524 bp) (NCBI Build 36.1; UCSC hg18, March 2006) across the human genome. Pooled male or female human commercial DNA samples were used as reference (Promega, Madison, WI, USA). Sample preparation and hybridization were performed following the NimbleGen CGH array standard protocol. Briefly, 500 ng of gDNA from each patient and sex-matched reference sample were labeled in parallel with Cy3 and Cy5, respectively. Subsequently, 20 µg of each labeled test and the corresponding sex-matched reference DNA were mixed and co-hybridized to the microarray overnight at 42°C. After hybridization, slides were washed and scanned at 2-µm resolution using the NimbleGen MS 200 Microarray Scanner (Roche NimbleGen) [2]. Raw data were extracted from the scanned images, processed and analyzed with NimbleScan software (version2.6; Roche NimbleGen). According to manufacturer’s recommendations and as quality controls during raw data processing, Grid Alignment, Sample Tracking Controls and Experimental Metrics Reports were evaluated by using objective scores and visual inspection. Then, aCGH data were analyzed with the segMNT algorithm from the NimbleScan software, which divides aCGH data into segments consisting of regions of homogenous copy number. It produces a variable called mad1.dr, which represents the median absolute deviation of the log_2_ ratio difference between consecutive probes along the chromosome. This parameter was used as a measure of the noise in the hybridization and to create sample-specific cut-offs for the detection of CNAs. An objective cut-off value above (gains) and below (losses) the mad1.dr parameter and a simultaneous visual examination of whole-genome view ratio plots using SignalMap (version1.9; Roche NimbleGen) were used to distinguish regions of gain or loss. Only CNAs larger than 500 kb were considered. Genome location and size of all CNAs detected by aCGH were converted to hg38 assembly (GRCh Build 38; UCSC hg38, December 2013) (<http://genome.ucsc.edu/cgi-bin/hgLiftOver>).

**Next-generation sequencing studies**

We applied amplicon-based next-generation sequencing using 454 Titanium amplicon chemistry (454 Life Sciences, Branford, CT, USA) in selected cases to investigate mutations occurring in *DNMT3A*, *TET2*, *RUNX1*, *TP53* and *BCOR* genes. The complete coding region of *TET2*, *RUNX1* and *BCOR,* exons 7-23 of *DNMT3A* and exons 4-11 of *TP53* were covered by 27, 7, 29, 16 and 8 amplicons, respectively (S3 Table). Amplicon libraries were prepared following the manufacturer’s recommendations and previously described methods [3]. In brief, individual amplicons were amplified in five preconfigured 96-well primer plates using the FastStart High Fidelity PCR System kit and GC-RICH PCR System kit (Roche Applied Science, Mannheim, Germany). After amplification, all amplicons of each plate were individually purified using Agencourt AMPure XP beads (Beckman Coulter, Krefeld, Germany), in order to remove short fragments, quantified with the Quant-iT PicoGreen dsDNA kit (Invitrogen, Carlsbad, CA, USA), adjusted to a final concentration of 1x10^9^ molecules/µL and combined in an equimolar ratio to generate the corresponding amplicon pools. Subsequently, each pool was adjusted to a final concentration of 2x10^6^ molecules/µL. Further, 454 Life Sciences NGS steps, such as the emulsion PCR, breaking of the emulsions, enrichment of beads carrying amplified DNA, loading DNA beads on a PicoTiterPlate and sequencing were performed following the manufacturer’s recommendations (454 Life Sciences). Multiple molecular barcodes were used to identify each patient. Gene and transcript IDs, information on primer sequences and the respective amplification mixes and cycler protocols are given below (S2, S3, S4 and S5 Tables).

All sequencing data were generated during several runs on the 454 Sequencing Systems, GS FLX and GS Junior, and processed using GS Run Browser software (version 2.9; 454 Life Sciences). All amplicon reads were aligned against the reference transcripts (S2 Table) of the corresponding genes (*DNMT3A*, *TET2*, *RUNX1*, *TP53* and *BCOR*) for variant detection using the Sequence Pilot software (version 3.5.2; JSI medical systems, Kippenheim, Germany) and GS Amplicon Variant Analyzer Software (version 2.9; 454 Life Sciences). For the detection of variants, filters were set to display sequence variants occurring in >2% of bidirectional reads per amplicon in at least one patient [3].

**References**

**1**. Vardiman JW, Thiele J, Arber DA, Brunning RD, Borowitz MJ, Porwit A, et al. The 2008 revision of the World Health Organization (WHO) classification of myeloid neoplasms and acute leukemia: Rationale and important changes. Blood. 2009;114(5):937-51. doi: 10.1182/blood-2009-03-209262.

**2**. Robledo C, García JL, Benito R, Flores T, Mollejo M, Martínez-Climent JÁ, et al. Molecular characterization of the region 7q22.1 in splenic marginal zone lymphomas. PLoS One. 2011;6(9):e24939. doi: 10.1371/journal.pone.0024939.

**3**. Kohlmann A, Klein HU, Weissmann S, Bresolin S, Chaplin T, Cuppens H, et al. The Interlaboratory RObustness of Next-generation sequencing (IRON) study: A deep sequencing investigation of TET2, CBL and KRAS mutations by an international consortium involving 10 laboratories. Leukemia. 2011;25(12):1840-8. doi: 10.1038/leu.2011.155.
